# Effects of Myosin “Essential” Light Chain A1 on the Aggregation Properties of the Myosin Head

**Published:** 2010-07

**Authors:** D.I. Markov, O.P. Nikolaeva, D.I. Levitsky

**Affiliations:** Bach Institute of Biochemistry, Russian Academy of Sciences; Belozersky Institute of Physico-Chemical Biology, Lomonosov Moscow State University

**Keywords:** myosin, subfragment 1, “essential” light chains, aggregation, thermal denaturation, differential scanning calorimetry

## Abstract

We compared the thermal aggregation properties of two isoforms of the isolated myosin head (myosin subfragment 1, S1) containing different “essential” (or “alkali”) light chains, A1 or A2. Temperature dependencies for the aggregation of these two S1 isoforms, as measured by the increase in turbidity, were compared with the temperature dependencies of their thermal denaturation obtained from differential scanning calorimetry (DSC) experiments. At relatively high ionic strength (in the presence of 100 mM KCl) close to its physiological values in muscle fibers, we have found no appreciable difference between the two S1 isoforms in their thermally induced aggregation. Under these conditions, the aggregation of both S1 isoforms was independent of the protein concentration and resulted from their irreversible denaturation, which led to the cohesion of denatured S1 molecules. In contrast, a significant difference between these S1 isoforms was revealed in their aggregation measured at low ionic strength. Under these conditions, the aggregation of S1 containing a light chain A1 (but not A2) was strongly dependent on protein concentration, the increase of which (from 0.125 to 2.0 mg/ml) shifted the aggregation curve by ~10 degrees towards the lower temperatures. It has been concluded that the aggregation properties of this S1 isoform at low ionic strength is basically determined by intermolecular interactions of the N–terminal extension of the A1 light chain (which is absent in the A2 light chain) with other S1 molecules. These interactions seem to be independent of the S1 thermal denaturation, and they may take place even at low temperature.

## INTRODUCTION

Cyclic interaction of the heads of myosin molecules with actin filaments accompanied by ATP hydrolysis underlies the molecular mechanism of biological motility in its various forms (from the events of intracellular transport to muscle contraction). It has been revealed that the myosin head is an example of a molecular motor which is able to fulfill its functions even when isolated [1]. A single myosin head, which is usually referred to as subfragment 1 (S1), is composed of two major structural domains known as the motor (or catalytic) domain and the regulatory domain. The motor domain is a globular structure containing both the ATPase active site and actin–binding site, whereas the regulatory domain is a long α –helix stabilized by noncovalent interactions with two other polypeptides, which are also known as essential and regulatory myosin light chains [2]. The present concept of the myosin motor function includes the rotation of the regulatory domain relative to the motor domain. During this rotation, the regulatory domain acts as a “lever arm” which amplifies and transmits conformational changes occurring in the motor domain during ATP hydrolysis. It has also been shown that the length of the “lever arm” (i.e., the regulatory domain) affects the amplitude of myosin head movement along the actin filament [3, 4].

The essential light chains associated with the regulatory domain of the myosin head are known to have two isoforms (a “long” one and a “short” one). Myosin from the cardiac muscle contains only the long light chain, whereas in a smooth muscle only the short chain is present. In fast skeletal muscle there are two kinds of the light chains, usually referred to as alkali light chains and designated A1 and A2 for the long and the short isoforms, respectively. These light chains are nearly identical, with the only exception being an additional N–terminal sequence of extra 41 residues present in A1 isoform. This N–terminal extension contains multiple Ala–Pro repeats, as well as some lysine residues [5]. The presence of the N–terminal extension remains unclear in terms of function and is subjected to extensive investigation. For example, it has recently been shown that mutations in this region tend to be associated with a type of severe congenital disorder known as hypertrophic cardiomyopathy [6].

S1 prepared by the chymotryptic digestion of skeletal–muscle myosin lacks the regulatory light chain but does contain the essential light chain [7]. Since the myosin of skeletal muscles contains alkali chains of both types, such an S1 preparation is essentially a mixture of myosin heavy chains complexed with either A1 or A2 (S1(A1) and S1(A2), respectively). These S1 species can be separated by means of ion–exchange chromatography [7] and used for a comparative functional analysis of A1 and A2 light chains, as well as for investigating the role of the N–terminal extension in A1. It was shown that, at low ionic strength, the S1(A1) affinity to actin greatly exceeds that of S1(A2) [8, 9] and N–terminal extension is involved in an additional interaction of A1 with actin filaments [10–13]. It is noteworthy that this interaction is merely observed at a low ionic strength, which is far from its physiological value and is shown to decrease markedly at 120 mM ionic strength [9].

Another intriguing feature of A1 N–terminal extension is its putative ability to interact with the globular motor domain of the myosin head. The possibility of this interaction was suggested by one of us more than 15 years ago [14] and was subsequently confirmed in works by other authors [15–17]. One recent study has revealed an interaction between the A1 N–terminal extension and the SH3 domain located near the N–terminus of the heavy chain (residues 35–80) [17]. The authors hypothesize that such a binding might play a significant role in the actin–myosin interaction, facilitating the straightening of the N–terminal extension into an antenna–like structure which is able to reach the surface of the actin filament.

Another interesting difference between the two S1 isoforms was revealed in earlier studies. Namely, it was shown that, at low ionic strength, S1(A1) aggregates at a substantially lower temperature than S1(A2) [18, 19]. It seems possible that, due to its semirigid extended structure, the A1 N–terminal segment can participate not only in intramolecular interactions, but also in intermolecular interactions with the motor domains of other S1 molecules. However, it should be noted that all previous experiments on S1 isoforms aggregation were carried out at very low ionic strengths and high protein concentrations [18, 19]. Unfortunately, nobody has undertaken a more thorough investigation of the thermal aggregation of S1 isoforms and the role of A1 N–terminal extension in this process. Therefore, a reasonable question arises: can intermolecular (or intramolecular) interactions of A1 N–terminal extension with the S1 motor domain affect S1 thermal aggregation at nearly physiological values of ionic strength? This is not a straightforward question, since a combined preparation of two S1 isoforms undergoes intensive thermal aggregation at the heat shock temperature (43°C) under salt conditions close to those in muscle fiber (100 mM KCl) [21]. In order to answer this question, in this study we performed a comparative analysis of the temperature dependencies of S1(A1) and S1(A2) aggregation at various ionic strengths and protein concentrations. We also compared the S1 thermal aggregation profiles with the temperature dependencies of its thermal denaturation obtained by differential scanning calorimetry (DSC).

## EXPERIMENTS

S1 was prepared by the digestion of rabbit skeletal myosin with α –chymotrypsin [7]. S1(A1) and S1(A2) preparations were obtained by ion exchange chromatography on a column of SP–trisacryl [22]. S1 concentration was estimated spectrophotometrically using the extinction coefficient E^1%^ at 280 nm of 7.5 cm^–1^. The absorption spectra of S1 isoforms were recorded on a Cary–100 spectrophotometer (Varian Inc.).

The temperature dependencies of S1–isoform aggregation were registered as an increase in the apparent optical density at 350 nm. The measurements were conducted on a Cary–100 spectrophotometer (Varian Inc.) equipped with a Biomelt thermostatted cell holder. The S1 samples were heated at a constant rate of 1 ° C/min from 25 ° C up to 65 ° C. All measurements were carried out in a 20 mM Hepes–KOH buffer (pH 7.3) containing 1 mM MgCl_2_ in the presence or absence of 10 0 m M KCl.

Thermal denaturation studies on S1(A1) and S1(A2) were carried out by means of DSC on a DASM–4M **** differential scanning microcalorimeter (Institute for Biological Instrumentation, Russian Academy of Sciences (RAS), Pushchino, Russia) as described earlier [21, 23, 24]. Samples containing S1 isoforms (1.5 mg/ml) were heated at a 1 ^o^C/min rate from 1 5 °C to 7 5 ° C in a 20 mM Hepes–KOH (pH 7.3) containing 1 m M MgCl_2_ in the presence or absence of 100 mM KCl. In order to check the reversibility of thermal denaturation after the first scan and subsequent cooling, protein samples were reheated. The thermal denaturation of both S1 isoforms was fully irreversible.

## RESULTS AND DISCUSSION

**Fig. 1:**
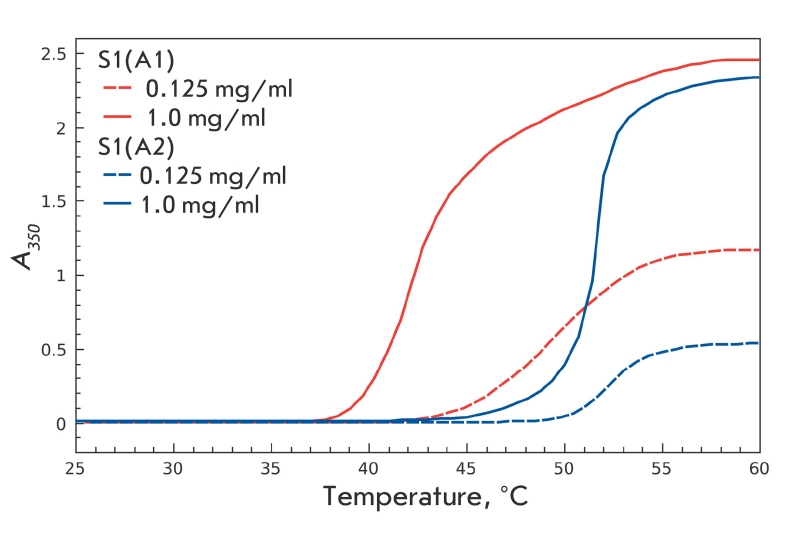
Temperature dependencies of the S1(A1) and S1(A2) thermal aggregations measured as an increase in apparent opti- cal density at 350 nm at high (1 mg/ml) and low (0.125 mg/ml) protein concentrations. Other conditions are as follows: 20 mM Hepes, pH 7.3, 1 mM MgCl_2_.

First of all, we were able to reproduce our longstanding results [19] comparing the thermal aggregation profiles of the two S1 isoforms at a high protein concentration (1 mg/ml) and a low ionic strength (in the absence of KCl). Fig. 1 shows that, under these conditions, the S1 isoforms substantially differ in the character of their thermal aggregation: S1(A1) aggregates at a much lower temperature than S1(A2) does. This difference between the isoforms becomes less pronounced at lower protein concentrations as is seen in Fig.1. Under these conditions, the half–maximum of increase in optical density for S1(A2) remains nearly the same (52–53 °C), while this parameter for S1(A1) shifts from 42.5 to 50 °C as the protein concentration is decreased from 1 mg/ml to 0.125 mg/ml. Thus, a decrease in protein concentration at low ionic strength strongly affects S1(A1) thermal aggregation. At the same time, the thermal aggregation of S1(A2) does not exhibit a strong dependence on protein concentration.

In subsequent experiments, we compared the normalized temperature dependencies of S1(A1) and S1(A2) aggregation obtained at different protein concentrations in the absence or presence of 100 mM KCl with the DSC profiles, which reflect thermal denaturation of the S1 isoforms under the same conditions. It is important to note that all the experiments were performed at the same heating rate (1 °C/min) and under similar salt conditions. However, the protein concentration remained constant (1.5 mg/ml) in DSC experiments, since earlier it had been shown that the variation in the protein concentration in the range of 0.5–2.0 mg/ml does not affect the temperature maximum of S1 heat–sorption curves [19]. Therefore, a comparison of the temperature dependencies of thermal denaturation and aggregation seems reasonable.

**Fig. 2:**
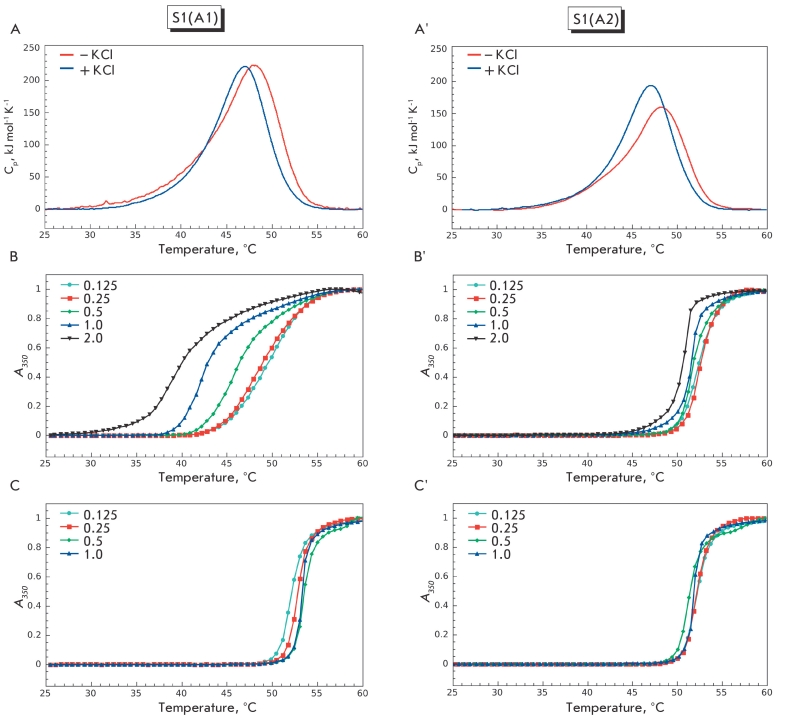
Fig. 2. Thermal denaturation and aggregation of isoforms S1(A1) (A–C) and S1(A2) (A`–C`). (A, A`) DSC curves obtained in the presence or absence of 100 mM KCl. (B, B`, C, C`) normalized temperature dependences of thermal aggregation of S1 isoforms ob- tained at various protein concentrations marked in each plot in the absence of KCl (B, B`) or in the presence of 100 mM KCl (C, C`). All experiments were performed at a heating rate of 1 °C/min. Other conditions are as follows: 20 mM Hepes, pH 7.3, 1 mM MgCl_2_.

The addition of KCl did not appreciably affect the thermal denaturation of both S1 isoforms, shifting the temperature maximum of the heat–sorption curve by 1.1 °C towards lower temperatures (from 48 to 46.9 °C in the case of S1(A1) or from 48.1 to 47 °C in the case of S1(A2); see Figs. 2A, 2A’). On the contrary, the salt concentration largely affected the thermal aggregation profile of S1(A1) but not S1(A2). If there was a low ionic strength, we observed a clear dependence of aggregation on the S1(A1) concentration. When the concentration increased from 0.125 to 2.0 mg/ml, the aggregation curve shifted by ~10°C towards lower temperatures (from ~ 50 to ~ 40 °C; Fig. 2B). Such effects were not observed in the presence of 100 mM KCl. In this case, the thermal aggregation of S1(A1) was almost independent of protein concentration: when the concentration of S1(A1) increased from 0.125 to 1 .0 mg/m l, the temperature of the half–maximum increase in optical density was constant and equal to 52 ± 0.5 °C (Fig. 2C). In the case of S1(A2), heat aggregation was independent of both protein concentration and ionic strength (Figs. 2B', 2C') and did not differ from S1(A1) aggregation at a high ionic strength (Fig. 2C).

From a comparison of thermal aggregation curves for S1 isoforms and the DSC profiles, which reflect their thermal denaturation, some conclusions can be drawn. First of all, the aggregation of S1(A2) is a result of its thermal denaturation. It seems possible that thermal denaturation of its more thermostable motor domain [14, 19] is responsible for the aggregation, since the denaturation of this domain has been shown to limit the aggregation of S1(A2). This is also applicable to the S1(A1) thermal aggregation at a high ionic strength (Fig. 2C). However, S1(A1) aggregation at a low ionic strength appears to be different (Fig. 2B), because it is characterized by the absence of any correlations between S1(A1) aggregation and denaturation. We assume that under these conditions S1(A1) aggregation is not determined by the protein thermal denaturation and can be at least partially explained by additional interactions between the A1 N–terminal extension and other S1 molecules. Obviously, the probability of such interactions must increase at higher protein concentrations and higher temperatures. Therefore, this could explain the unusual aggregation profile observed in the case of S1(A1) at low ionic strength (Fig. 2B). At high ionic strength, the intermolecular interactions of the A1 N–terminal extension should be weakened, which explains the observed similarity between the S1(A1) and S1(A2) aggregation profiles in the presence of 100 mM KCl (Fig. 2C, 2C').

**Fig. 3:**
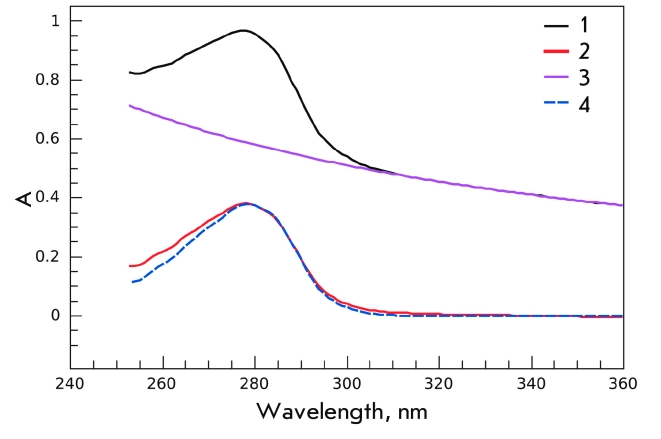
Fig. 3. Absorption spectra of a S1(A1) preparation (1 mg/ml), stored at 4 °C at low ionic strength (20 mM Hepes, pH 7.3, 1 mM MgCl_2_) measured before (1) and after (2) addition of KCl up to a concentration of 100 mM. Curve 3 was obtained by extrapolation of the long-wavelength part of S1(A1) absorption spectrum into its short- wavelength region, and it reflects light-scattering of the S1(A1) preparation at low ionic strength in the entire wavelength range. Curve 4 was obtained by subtracting curve 3 from curve 1.

When thoroughly analyzing the S1(A1) aggregation curves obtained at low ionic strength (Fig. 2B), one may notice that, at high protein concentrations, aggregation starts at relatively low temperatures (below 38 °C). Therefore, we can suggest that, at low ionic strength, S1(A1) aggregation based on the intermolecular interactions of the A1 N–terminal extension can occur slowly at a low temperature. Actually, we have observed noticeable opalescence in S1(A1) preparations which disappeared after the addition of 100 mM KCl. (It is noteworthy that, in thermal aggregation experiments, these opalescent S1(A1) preparations had been preliminarily subjected to ultracentrifugation.) These observations were confirmed by experimental results shown in Fig. 3. As is seen, keeping the S1(A1) preparation overnight at 4 °C leads to an increase in light scattering in the range of 320–360 nm, i.e. where proteins do not absorb (Fig.3, curve * 1 * ). The opalescence fully disappears after the addition of 100 mM KCl (Fig.3, curve * 2* ). Extrapolating from the high wavelength range of the S1(A1) absorption spectrum, we were able to deduce the wavelength dependence of the sample’s light scattering within the whole range of wavelengths (255–360 nm). Subtracting this curve (Fig. 3, curve * 3 * ) from the measured S1(A1) absorption spectra yielded curve * 4 * , which corresponds to the S1(A1) spectra with no impact of light scattering. The latter was indistinguishable from the S1(A1) spectra measured in the presence of 100 mM KCl (Fig. 3, curve * 2* ).

The results of this experiment clearly show that S1(A1) aggregation based on intermolecular interactions of the A1 N–terminal extension at low ionic strength can even take place during the storage of an S1(A1) preparation in a fridge. This aggregation is reversible, because the forming aggregates can be easily dissolved at a high ionic strength. At this point, the reversible aggregation strongly differs from thermal denaturation–induced irreversible aggregation, which is accompanied by the cohesion of denatured protein molecules.

Therefore, the described experiments lead to the conclusion that the difference in the aggregation properties of the S1 isoforms is based on an additional interaction between the A1 N–terminal extension, which is absent in A2 light chain, and other S1 molecules. These interactions occur only at low ionic strength and are suppressed at a high ionic strength. These interactions take place even at a low temperature, though the probability of their formation increases at higher temperatures. To all appearances, these intermolecular interactions reflect the ability of the A1 N–terminal extension to bind to the motor domain of the same S1 molecule. Such an interaction is supposed to play an important role in the mechanism of muscle contraction [16, 17]. However, it should be noted that all previous studies on the intramolecular interactions of the A1 myosin light chain were performed at a low ionic strength (~25 mM) [17], which is far from its physiological values. We can suggest that the probability of these intramolecular interactions should increase during the ATPase reaction. This could be due to the A1 N–terminal extension being brought into close proximity with the S1 motor domain, which could possibly occur as a consequence of the rotation of the regulatory domain relative to the motor domain. This, in turn, would decrease the probability of intermolecular interactions of the A1 N–terminal segment, which should affect the aggregation properties of S1(A1) when intermediate states of the ATPase reaction are modeled in an experiment. These assumptions need to be experimentally confirmed, which is among the goals of future studies in this field.

## CONCLUSIONS

In this study we have shown that, at a relatively high ionic strength (close to that in the muscle fiber), the presence of an additional N–terminal segment in the myosin A1 light chain does not affect the aggregation properties of the isolated myosin head (S1). Under these conditions, S1 thermal aggregation follows its thermal denaturation and is caused by the cohesion of denatured protein molecules. A noticeable influence of the A1 N–terminal segment on the S1 aggregation is observed only at a relatively low ionic strength. Under these conditions, the intermolecular interactions of the A1 N–terminal extension appear to be the main factor underlying the aggregation properties of S1. These intermolecular interactions of the A1 N–terminal segment reflect its ability to form intramolecular interactions, which are thought to play an important role in muscle contraction. Presumably, under certain conditions (e.g., during the ATPase reaction, which is accompanied by considerable conformational changes in the myosin head), intramolecular interactions of the A1 N–terminal segment can take place in muscle fibers even at a relatively high ionic strength.
